# Editorial: Advanced application technology for plant protection: Sensing, modelling, spraying system and equipment

**DOI:** 10.3389/fpls.2023.1113359

**Published:** 2023-02-23

**Authors:** Changyuan Zhai, Wei Qiu, Paul Weckler, Xiongkui He, Khawar Jabran

**Affiliations:** ^1^ Intelligent Equipment Research Center, Beijing Academy of Agricultural and Forestry Sciences, Beijing, China; ^2^ College of Engineering, Nanjing Agricultural University, Nanjing, China; ^3^ Department of Biosystems & Agricultural Engineering, Oklahoma State University, Stillwater, OK, United States; ^4^ Centre for Chemicals Application Technology (CCAT), China Agricultural University, Beijing, China; ^5^ Department of Plant Production and Technologies, Niğde Ömer Halisdemir University, Niğde, Türkiye

**Keywords:** unmanned aerial vehicles, drift characteristics, deep learning, pests and diseases, airflow velocity loss characteristics, air-assisted spray

As editors of this Research Topic, we summarized the contributions of 20 articles accepted in this topic. The research contents mainly focused on the following sections: drift characteristics of unmanned equipment and development of autonomous navigation spray system, identification and classification of pests and diseases based on deep learning, and airflow velocity loss characteristics of air-assisted spray in orchard.

## Drift characteristics of unmanned equipment and development of autonomous navigation spray system

In recent ten years, agricultural unmanned aerial vehicles (UAV), also known as Unmanned Aerial Spraying Systems (UASS), as a new method for the application of plant protection products, has developed rapidly in the world. Compared with ground equipment, UAV spray is more likely to cause spray drift and environmental pollution to non-target areas. Therefore, it is important to study the spray drift characteristics of UASS.

Chen et al. reviewed the drift characteristics of UAV spray system and the factors affecting UAV system drift, and put forward suggestions on the optimization of spray system and structure layout, modeling of drift test, and standardization of measurement methods. Jiang et al. compared the performance of UAV, unmanned ground vehicle (UGV) and spray gun pesticide application technology of spray target coverage, off-target coverage, time efficiency and gasoline consumption in the pear orchard. The results showed that compared with UGV, UAV has the advantages of high working efficiency, less environmental pollution and consumption of natural resources. Although the traditional spray gun technology showed good spraying performance, it is not conducive to the protection of environment and resources. The achievement was helpful to the research and development of intelligent pesticide application technology. Shan et al. carried out research on corn fertilization method based on UAV. The difference of the effectiveness between the water sprayed on the sampling rod and leaves was studied. They found that sampling methods have a significant effect on deposition results and determined the optimum spraying concentration. Qi et al. compared the effects of multi-functional unmanned aerial vehicle (mUAV) planter, mechanical rice seeder and mechanical rice transplanter on rice cultivation. The results showed that there was no significant difference in rice yield among the three regions. In terms of labor cost and seeding efficiency, UAV was more effective than mechanical planter and transplanter. Li et al. studied the influence of UAV flight velocity on deposition distribution and droplet size, especially the usage of compound pesticides as spray solution. The results demonstrated that increasing flight velocity is helpful for pesticide droplets to spread and penetrate the canopy. However, it also led to uneven deposition of pesticides, reduced deposition volume, and reduced effective coverage and effective density ratio.

Jiang et al. developed a greenhouse autonomous navigation system based on Simultaneous Localization and Mapping (SLAM) algorithm. In this paper, three-dimensional Lidar data was filtered and fused into two-dimensional Lidar data containing the environment information in the range of robot motion height. They used Dijkstra algorithm for global planning and DWA algorithm for local navigation path planning of robot. This method not only ensured the accuracy of greenhouse environment map but also reduced the accuracy of greenhouse environment map and the performance requirements of industrial computers. According to the three perception decision control modules of unmanned system, Wang et al. constructed the environment perception and map building strategy based on 3D Lidar under the complex environment background of orchard. They pointed out two difficulties in developing automatic orchard sprayer: one is to realize efficient penetration of pesticides in low-density canopy and reduce losses, and the other is to make the machine automatically pass through orchard without manual control. They also provided a basis for the development of technology for independent and precise spraying of pesticides in the orchard environment based on automatic navigation.

## Identification and classification of pests and diseases based on deep learning

At present, researches have carried out extensive research in the fields of pest image recognition, segmentation and feature extraction based on deep learning. The purpose of most researches was to improve the running speed and recognition accuracy of the system by optimizing or building the models.

Zhao et al. proposed an improved deep convolution neural network to identify crop pests. They also developed a new attention module, which includes parallel attention mechanism module and residual blocks. This module was integrated into ResNet-50 CNN, which is used to classify 10 different types of crop pests. This network had significant advantages in terms of accuracy and real-time performance compared with other models. Yao et al. studied the segmentation and recognition of peach disease based on Mask R-CNN and Mask Scoring R-CNN to provide evidence for disease control and treatment. This work was valuable in engineering applications, such as the classification of plant diseases and the location and segmentation of lesion areas. Li et al. proposed an imaging model for detecting corpuscle insects such as whitefly and thrips in greenhouse. The author used an automatic detection method to reduce pest detection. This method could satisfy the needs of continuous monitoring of pests in greenhouse, and estimate the total population density. Lin et al. proposed a few-shot learning method for plant disease recognition based on multi-scale feature fusion and attention. The results showed that plant disease identification technology based on a few-shot learning method is feasible in the future application.

## Airflow velocity loss characteristics of air-assisted spray in orchard

Air-assisted spray technology has been widely used in the high-efficiency application of pesticides in orchards. In this section, the authors mainly studied the influence characteristics of canopy airflow velocity loss on air-assisted spray performance.

Zhang et al. established a theoretical model of airflow velocity attenuation in a pear canopy by selecting the velocity attenuation factor k and incoming velocity as model inputs. It was demonstrated that high-speed airflow will disturb the outer branches and leaves and thus affecting the accuracy of the model. The research results could provide theoretical basis for the adjustment of air flow parameters of air-assisted spray in the pear orchard. Wu et al. discussed the feasibility of using the resistance characteristics of crop canopy to evaluate its droplet deposition effect through theoretical analysis and wind tunnel test. The results could provide theoretical basis for rapid and low-cost research and development of crop protection technology and equipment. Yang et al. proposed a new three-dimensional airflow velocity and direction synchronous measurement method, and established a new sensor system and calculation model. This method could be used as a solution to measure and evaluate the airflow velocity field characteristics of sprayers. Gu et al. studied the airflow velocity loss model for a canopy. They built a three-dimensional airflow velocity measurement platform for fruit tree canopy, and obtained the point cloud data by Lidar scanning. Classical regression, partial least squares regression (PLSR) and back propagation (BP) neural network algorithms were adopted. This study could provide a basis for airflow velocity control of precise variable spray and promote the development of airflow velocity control technologies.

In addition to these three main sections, the authors also conducted the following researches. Zhang et al. proposed a method to evaluate the adjuvant efficacy of herbicides under different temperature conditions by using chlorophyll fluorescence of herbaceous plants. The experiment was carried out under the control of greenhouse environment by using two-factor block experiment scheme. The results distinguished the differences among treatments and determined the optimum solution to improve the efficacy of topramzone against weeds at different temperatures. Electrochemical fingerprinting technology can collect the electrochemical behavior of electrochemically active molecules in plant tissues, which is considered as a new plant analysis technology. Hu et al. found that electrochemical fingerprint signals are positively correlated with the number and type of electrochemical active molecules in plant tissues, and can also be used to reflect the genetic differences among different species. Liu et al. established the numerical model of plant-soil-machine system, and introduced the details on the construction and calibration method of plant mechanics model based on the discrete element method (DEM). The discrete element model of taro plant established in this paper was reliable. In order to solve the issues of low weeding rate and severe seedling damage of rice weeding machinery, Zhang et al. optimized the key components of rice weeding. Through the analysis of the motion trajectory and DEM simulation analysis of the weeding wheel, the structural parameters of the weeding wheel were determined. This study provided a technical reference for the improvement of paddy-field weeding equipment. Xie et al. proposed a new method to predict the waterlogging tolerance of poplar. They used different feature selection algorithms to analyze the waterlogging tolerance of different parameters such as photosynthesis and chlorophyll fluorescence. Machine learning algorithm was used to study and analyze different parameters of poplar waterlogging resistance. This research provided new information for the selection of poplar seedlings with waterlogging tolerance.

## Author contributions

All authors listed have made a substantial, direct, and intellectual contribution to the work and approved it for publication.

